# Record of massive upwellings from the Pacific large low shear velocity province

**DOI:** 10.1038/ncomms13309

**Published:** 2016-11-08

**Authors:** Pilar Madrigal, Esteban Gazel, Kennet E. Flores, Michael Bizimis, Brian Jicha

**Affiliations:** 1 Department of Geosciences, Virginia Tech, Blacksburg, Virginia 24061, USA; 2 Department of Earth and Environmental Sciences, Brooklyn College of the City University of New York, Brooklyn, New York 11210, USA; 3 Department of Earth and Planetary Sciences, American Museum of Natural History, New York, New York 10024, USA; 4 Department of Earth and Ocean Sciences, University of South Carolina, Columbia, South Carolina 29208, USA; 5 Department of Geoscience, University of Wisconsin–Madison, Madison, Wisconsin 53706, USA

## Abstract

Large igneous provinces, as the surface expression of deep mantle processes, play a key role in the evolution of the planet. Here we analyse the geochemical record and timing of the Pacific Ocean Large Igneous Provinces and preserved accreted terranes to reconstruct the history of pulses of mantle plume upwellings and their relation with a deep-rooted source like the Pacific large low-shear velocity Province during the Mid-Jurassic to Upper Cretaceous. Petrological modelling and geochemical data suggest the need of interaction between these deep-rooted upwellings and mid-ocean ridges in pulses separated by ∼10–20 Ma, to generate the massive volumes of melt preserved today as oceanic plateaus. These pulses impacted the marine biota resulting in episodes of anoxia and mass extinctions shortly after their eruption.

## Abstract (web summary)

Large igneous provinces may record pulses mantle plume upwellings and their relationship with deep-rooted mantle sources. Madrigal *et al*. present a new petrological model of the Pacific Ocean Large Igneous Province finding that mantle plume pulses were separated by 10–20 Ma.

## Graphical abstract

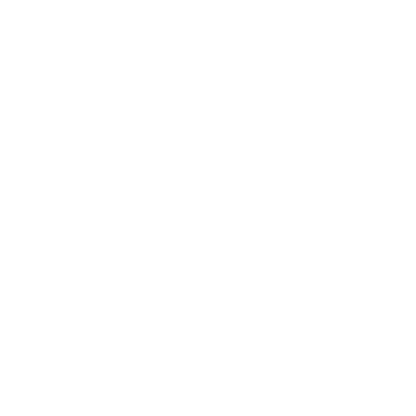

Global tomography and numerical models suggest that mantle plume occurrences are closely linked to the margins of large low-shear velocity provinces (LLSVPs)^1^,^2^,^3^,^4^. In these marginal zones the ascent of material from the core-mantle boundary connects deep mantle dynamics with surface processes through mantle plume activity, forming large igneous provinces (LIPs) and some of the modern hotspot volcanoes^5^,^6^. Petrological^7^ and geodynamic^8^ evidence suggest a link between the formation of oceanic plateaus and the interactions of mantle plumes and mid-ocean ridges (MORs). Even though the causality relationship between both processes is still unclear (either rifting is initiated by plume impact or it pre-dates plume interaction) larger volumes of upwelling mantle material will preferentially reach the surface when impacting or captured by a MOR^8^,^9^. Consequently, it is possible to trace the potential interactions between MORs and mantle plume upwellings by referencing the tectonic and magmatic evolution of the Pacific Plate in time to the current location of the LLSVP, considering the long-lived (∼500 Ma) existence of these thermochemical anomalies^1^,^8^,^10^.

Here, we identified episodic upwellings of the Pacific LLSVP during the Mesozoic by reconstructing the kinematic evolution of the Pacific Plate in the last ∼168 Ma using the record of LIP fragments, both accreted in tectonic margins and at the seafloor. To accurately reconstruct the paleo-Pacific Plate layout, we included both the oceanic plateaus and ocean-basin flood basalts, however for the purpose of this paper hereafter we will refer to both groups as oceanic LIPs considering that the processes that formed both types of features are intrinsically related^11^ as both are generated by extensive adiabatic decompression of material hotter than ambient asthenospheric mantle^11^,^12^.

## Results

### Record of Pacific related LIPS accreted in Costa Rica

**Figure 1:**
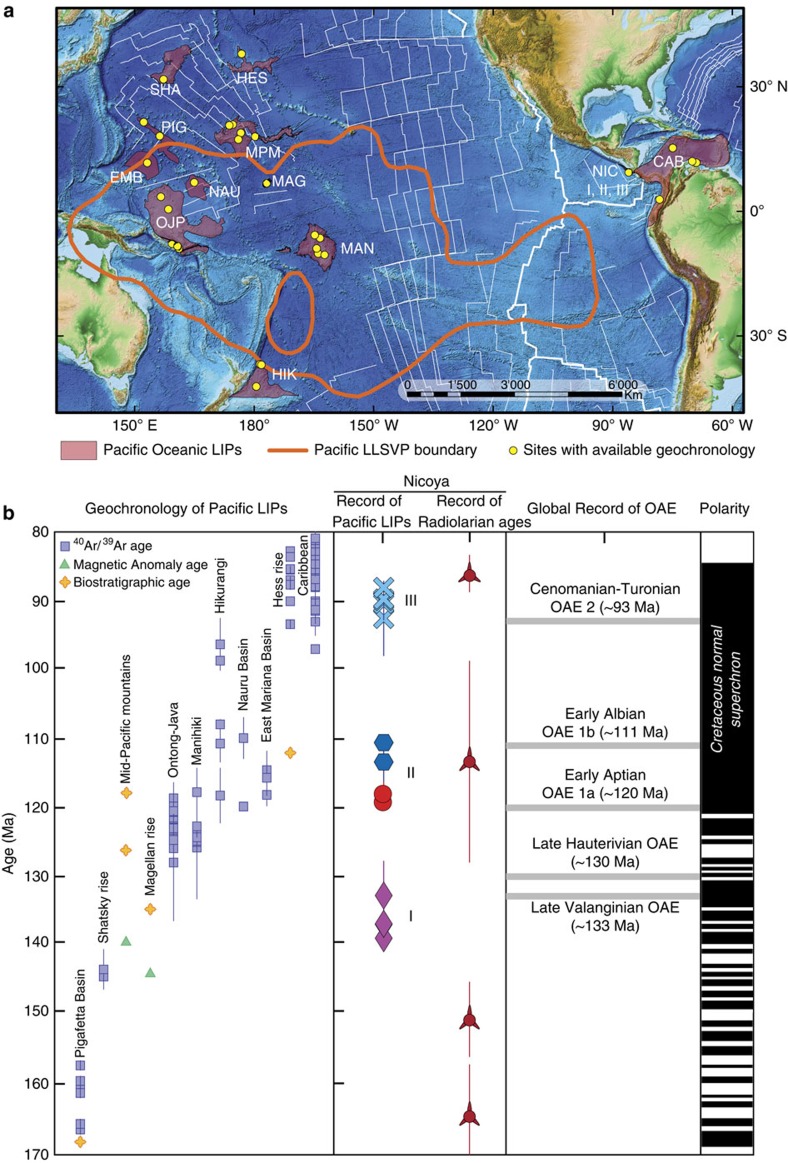
Current Pacific Plate LIPs location and ages. (**a**) Present configuration of the central circum-Pacific Plate; highlighted purple areas correspond to fragments of oceanic plateaus and ocean-basin flood basalts preserved at the seafloor and plate margins, yellow dots are sampled locations with geochronology and/or geochemistry, and the white lines represent magnetic anomalies of the Pacific seafloor after Muller^21^. The boundaries of the Pacific LLSVP (ref. 18) at 2500, km are projected to the surface. (**b**) Geochronological correlation of Pacific LIPs with the oceanic accreted terranes in Costa Rica (pillow basalts and radiolarites), global oceanic anoxic events^53^ and geomagnetic polarity reversals^56^. Purple diamonds denote the Nicoya pillow basalts at 140 Ma, red circles at 120 Ma, blue hexagons at 110 Ma, and blue exes at 90 Ma; three-pointed red symbols denote radiolarian ages with their respective error bars. CAB, Caribbean; EMB, East Mariana Basin; HES, Hess Rise; HIK, Hikurangi; MAG, Magellan Rise; MAN, Manihiki; MPM, Mid-Pacific Mountains; NAU, Nauru Basin; NIC I, Nicoya I; NIC II, Nicoya II; OJP, Ontong-Java; PIG, Pigafetta Basin; SHA, Shatsky Rise.

**Figure 2:**
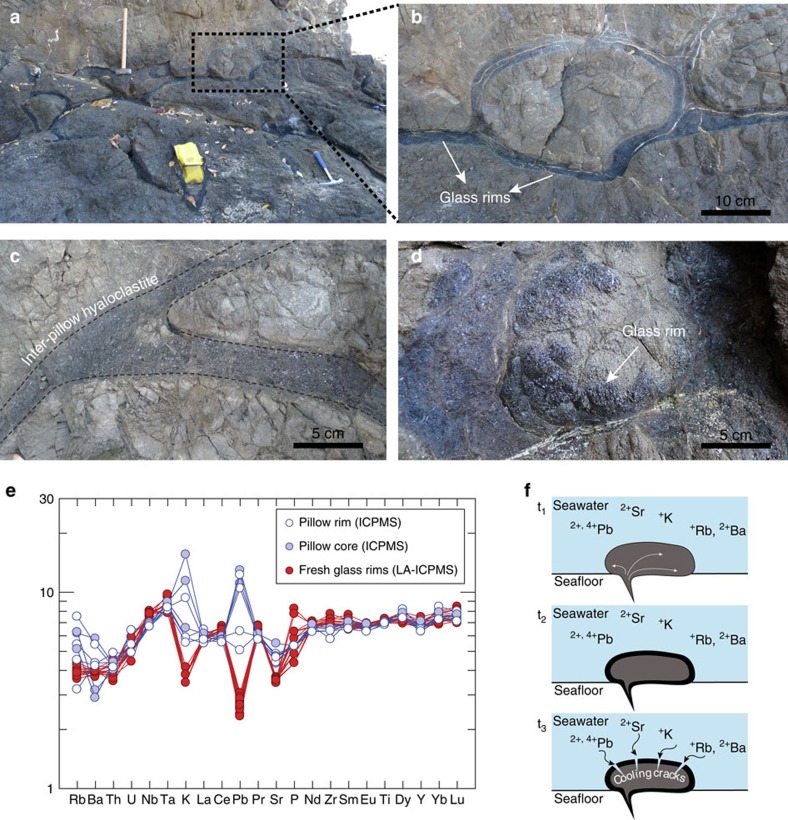
Field photographs of the pillow basalt outcrops at Nicoya Peninsula, Costa Rica. (**a**) Pillow basalt flows at San Juanillo beach (lat/long: 10.03020/-85.7394) with quenched glass rims surrounding the pillow basalts. (**b**) Closer look at an individual pillow basalt; the rims preserved glasses with little to no alteration. (**c**) The hyaloclastite between pillow basalts also contains fragments of fresh glasses. (**d**) Close up of the fresh glass rim surrounding the pillow basalt. (**e**) Multielement diagram showing the marked differences in fluid mobile element contents between analysis made *in situ* on pillow basalt glass rims (red symbols) by LA-ICP-MS and on whole-rock analyses from the pillow cores (blue closed symbols) and the pillow rims (blue open symbols). (**f**) Schematic representation of the seawater-pillow basalt interaction at eruption on the seafloor. At t_1_ the pillow basalt erupts (T∼1,000 °C) at the seafloor where immediately gets in contact with cold seawater (T∼1 °C). In t_2_, the thermic shock results in the instantaneous quenching of the lava, which creates a glass rim surrounding the pillow basalt. At t_3_ the pillow basalt starts to cool down and contracts, creating radial fractures that allow the entrainment of seawater.

LIPs emplaced over oceanic plates become thickened, buoyant sections of the lithosphere, making them prone to collision and accretion instead of subduction, as they reach convergent margins by means of the normal spreading processes of the oceanic plates^13^. These accretionary processes preserved a series of LIP fragments along the Pacific coast of Costa Rica (Supplementary Note 1 and Supplementary Fig. 1), that range from near the formation of the Pacific Plate at ∼170 Ma to the last Pacific LIP at ∼90 Ma, representing one of the most complete records of Pacific Plate mantle upwelling events that resulted in LIPs^14^,^15^,^16^ (Fig. 1a,b). The Nicoya Complex in north-western Pacific coast of Costa Rica (Fig. 1) groups a series of terranes of oceanic origin that include well-preserved pillow basalt flows (Fig. 2). Chronologically these suites of lavas represent three main events: Nicoya I at ∼140 Ma, Nicoya II at ∼120 Ma and Nicoya III at ∼90 Ma^15^ (Fig. 1 and Supplementary Table 1). The lavas preserve fresh pillow-rim glasses (Fig. 2), which we used to generate new geochronological and geochemical data from these oceanic LIP accreted terranes. These data were incorporated in a global compilation of radiometric (^40^Ar/^39^Ar), biostratigraphic and magnetic anomaly ages from Pacific LIPs (Supplementary data 1–6), and they reveal periods of enhanced magmatic activity associated with mantle plume upwellings, almost since the formation of the Pacific Plate (Fig. 1a,b). These Nicoya terranes were originally thought to belong to the Caribbean LIP^15^,^17^, however the ages of the Nicoya I and Nicoya II basalts are not consistent with a Caribbean LIP origin. Their geochemical signature and association with black shales that record global oceanic anoxic events (Fig. 1b) suggest that they represent fragments of older LIPs. Therefore, to better assess their provenance, we created a series of kinematic reconstructions to show the possible episodicity of the Pacific LLSVP upwellings as well as the possible location where Nicoya I and II formed.

### Kinematic model of the Pacific Plate and LIP formation

By superimposing the limits of the current Pacific LLSVP (ref. 18) on the surface of our kinematic plate tectonic reconstructions (details in the Methods section and Supplementary Figs 2–5) we can assess the potential interactions between the location of MORs and potential upwellings of the LLSVP in time (Fig. 1a). Many of the existent Pacific tectonic reconstructions used only the position of hotspots as a fixed reference frame; however, this could introduce inconsistencies due to hotspot movement in large time scales since plumes could be affected by mantle ‘wind' or could be captured by a MOR^19^,^20^. Therefore, to create a more accurate reconstruction in our kinematic model, we used an integral combination of paleomagnetic anomalies (reported^21^ and reconstructed) with seafloor geometry (MORs, magnetic anomalies and relative locations of hotspot), and the geological and kinematic evolution of the circum-Americas active margins.

**Figure 3:**
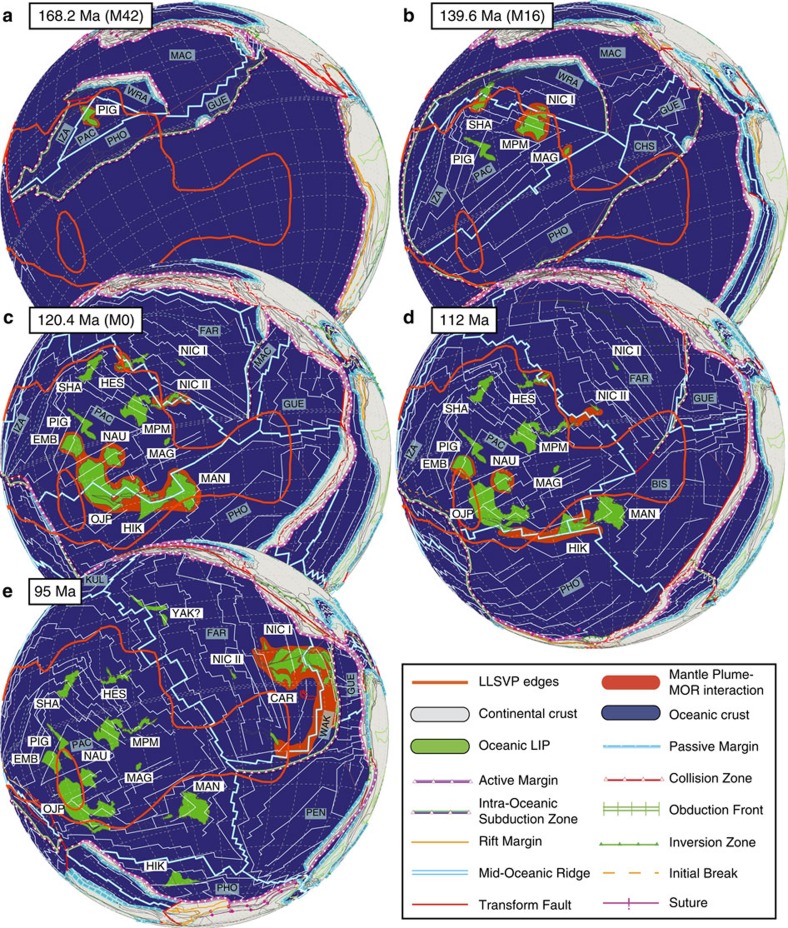
Kinematic plate tectonic reconstructions for circum-Pacific Plate. ‘M' notations refer to established magnetic anomalies. The orange outline denotes the Pacific LLSVP. MORs are represented by thick light blue lines and magnetic anomalies in white thin lines. (**a**) At ∼168.2 Ma (M42) the Pacific Plate was at its onset. Pigafetta Basin (PIG) was forming. (**b**) At ∼139.6 Ma (M16) a period of active upwellings of the Pacific LLSVP at its N-NE margins triggered the formation of Shatsky Rise (SHA) at circa 144 Ma, Nicoya I (NIC I) plateau and Mid-Pacific Mountains (MPM) at circa 140 Ma and Magellan Rise (MAG) at circa 135 Ma. (**c**) At ∼120.4 Ma (M0) a new period of mantle upwellings stimulated the formation the Ontong-Java (OJP), Manihiki (MAN) and Hikurangi (HIK) Plateau event. The Nicoya II (NIC II) plateau belongs to these series of upwellings, erupting close to the northern margins of the LLSVP. Also at this time, a rejuvenated stage occurred at the Mid-Pacific Mountains characterized by the formation of several sub-aerial seamounts with a clear OIB signature that were eroded and later subsided. Meanwhile, the East Mariana Basin (EMB), near the W margins of the Pacific LLSVP, presented an intraplate magmatic pulse at circa 127 Ma and 120 Ma, respectively. (**d**) At ∼112 Ma the southern margin of the LLSVP remains active and in interaction with a MOR, forming sections of the Hikurangi Plateau, Nauru Basin (NAU) and East Mariana Basin. (**e**) At ∼95 Ma the easternmost margins of the Pacific LLSVP became active forming the Caribbean Plateau (CAR) at the intersection of with a MOR. Tectonic plate abbreviations BIS (Biscoe), CHS (Chonos), FAR (Farallon), GUE (Guerrero), IZA (Izanagi), KUL (Kula), MAC (Mackinley), PAC (Pacific), PEN (Penas), PHO (Phoenix), WAK (Washikemba), WRA (Wrangellia) and YAK? (Yakutat).

Our model evidences a close relationship between the formation of Pacific LIPs, the paleo locations of MORs and the margins of the Pacific LLSVP at specific times. At ∼168 Ma our reconstruction shows the birth of the modern Pacific Plate and on its north-western border, close to the LLSVP margin, the emplacement of the Pigafetta Basin (Fig. 3a). This basin contains the oldest oceanic crust of the Pacific Plate (∼170–160 Ma^22^,^23^) and can be associated with a potential deep upwelling at the northwest edge of the LLSVP boundary. Even though the Pigafetta Basin is considered an ocean-basin floor basalt type of oceanic LIP, its formation could be related to the interaction of the mantle plume derived from the Pacific LLSVP and a MOR. This interpretation is in good agreement with the high resolution ^40^Ar/^39^Ar data collected by Koppers, *et al*.^24^ and with previous reconstruction models (Supplementary data 1).

Chronologically the next series of Pacific LIPs include the Nicoya I, Shatsky Rise and Magellan Rise at ∼140 Ma along with the plateau basement that contains the Mid-Pacific Mountains (Fig. 2b). In the ∼140 Ma reconstruction, the LIPs fit along a northern MOR that coincides with the LLSVP margin at that time, except from Magellan Rise, which forms at a MOR further to the southeast. The Mid-Pacific Mountains seat over an abnormally elevated seafloor area that have been interpreted as a mid-Cretaceous Superswell. The seamounts and guyots that constitute the Mid-Pacific Mountains, some of which have been drilled by DSDP and ODP cruises, display ^40^Ar/^39^Ar ages between 123 and 128 Ma^25^; however, the main bathymetric height that constitute their basement has not been successfully reached by drilling and its age is thought to be as old as Upper Jurassic-Lower Cretaceous^25^. The isotopic and geochemical enrichments of the alkali basalts recovered from these seamounts and guyots are in agreement with a volcanism controlled by shallow mantle processes and the tectonic stresses within the original plateau^26^. Hence, here we hypothesize that the Mid-Pacific Mountains represent a rejuvenated stage of a much older oceanic LIP that had its main volcanic constructing pulse circa 140 Ma^25^. This oceanic LIP appears in our ∼140 Ma reconstruction notably alongside the oldest suites of the Nicoya I terrane (Fig. 1b) accreted in Costa Rica. We propose that the Nicoya I oceanic terrane constitute a drifted piece of that LIP which was subsequently accreted to the coast of Costa Rica (Fig. 3b).

In the ∼120 Ma reconstruction a major episode of upwelling from the Pacific LLSVP generated the largest oceanic LIPs recorded in Earth's history: Ontong-Java, Manihiki and Hikurangi LIPs (Fig. 3c) which are thought to have erupted synchronously as a single plateau that was later rifted apart^27^,^28^,^29^,^30^. The East Mariana Basin and Nauru Basin oceanic LIPs also formed at this stage. These LIPs are possibly related to the formation of the Ontong-Java, Manihiki and Hikurangi Plateau event^27^ which could have extended laterally, creating a thinner structure than the main plateau event. Younger seamounts from rejuvenated stages on top of these LIPs record intra-plateau deformation and low degree of partial melting of shallow heterogeneities^27^, and thus are not representative of deep mantle upwellings as considered in other studies^31^. In our model, these LIPs formed along an east–west oriented MOR near the southern LLSVP margin. Likewise, at the northern edge of the LLSVP a potential upwelling reached a MOR at this stage. This upwelling triggered the eruption of the ca. 120 Ma basaltic suites that were accreted in the Nicoya Peninsula (Nicoya II, Fig. 1). We propose that the Nicoya II terrane formed alongside the younger section of the Mid-Pacific Mountains basement, but on the northwestern side of the MOR, causing the Nicoya II terrane to migrate towards the western margins of the Pacific Plate (Fig. 3c).

Our model is also in good agreement with chronological and geometrical data, which suggest that there is a section of the Hikurangi Plateau that is slightly younger than Ontong-Java and Manihiki^28^ (Fig. 3d). At the ∼112 Ma reconstruction the interactions between the southern margin of the LLSVP and a MOR triple junction separated the Hikurangi LIP from the other plateaus; this upwelling is likely responsible for forming the younger sections of the Hikurangi Plateau (Figs 1b and 3d). In the case of the Hess Rise, at the DSPD site 464 (ref. 32), sediments of Albian (∼113–100 Ma) age have been drilled, suggesting that the basement below could be older than the radiogenic ages of the recovered material. Our model shows that this basement was possibly formed at the northern limits of the LLSVP margin between 120 and 112 Ma (Fig. 3c,d). Ages from the Nicoya II terrane circa 110 Ma also suggest that active upwelling was taking place at the northern LLSVP edge at this time (Fig. 3d).

Interestingly, there is no record for Pacific Ocean related LIP formation at ∼103 Ma; however, our model suggests an interaction between a MOR and the northern edges of the LLSVP, potentially triggering the formation of an LIP at this time (Supplementary Fig. 3g). A plausible candidate could be the undated Yakutat oceanic terrane^33^, which initially collided with the North American margin, then translated along the margin and became emplaced at the southern Alaska margin^33^. Furthermore, this raises the possibility of the existence of upwelling pulses of the LLSVP that have not yet been identified in the geologic record.

The youngest Pacific LLSVP upwelling pulses at ∼90 Ma, include the Caribbean LIP and the younger parts of the Hess Rise. Recovered fragments of the Hess Rise of ∼90 Ma show clear geochemical signature consistent with a LIP^34^. At the ∼95 Ma reconstruction (Fig. 3e) the Caribbean Plateau is forming by a plume connected to the easternmost part of the LLSVP in a near equatorial location. Although the interaction between the LLSVP upwelling and the MOR is only evident in the southern region, we suggest that the plume was captured by the MOR^20^, hence the northward configuration of the LIP, and probably still interacting until ∼67 Ma (Supplementary Figs 3 and 4); a similar model has been proposed to explain the massive volume and configuration of the Ontong-Java, Manihiki and Hikurangi event^29^. Fragments of the Caribbean LIP have been identified in the Nicoya Peninsula as suites of massive basalts, diabase and gabbro intrusions. Radiometric ^40^Ar/^39^Ar ages group these suites of oceanic rocks between 92.5 and 83.2 Ma (Fig. 1b), consistent with the ages reported for the Caribbean Plateau in the region (Caribbean, Colombia, Ecuador; Fig. 1b and Supplementary data 1). Finally, during our ∼68 Ma and ∼56 Ma reconstruction (Supplementary Fig. 3j,k) the Caribbean LIP is positioned in between the Americas and as the plate kept moving towards the east, subduction initiation occurs in its western boundary, forming the early Central America Trench.

### Evidence for plume-ridge interaction in LIP formation

Immediately after basaltic lavas erupt in deep oceanic environments a quenching glass rim forms around the pillow basalt (Fig. 2a–d). Numerous authors have described the physical and chemical dynamic of such interaction, which usually results in an exchange of dissolved cations between seawater and the newly formed oceanic crust (Fig. 2e,f)^35^,^36^. During the quenching process, a decrease of volume occurs, allowing the development of several sets of radial cracks surrounding the pillow basalts. These cooling cracks enable the seawater to percolate inside the hotter interior of the pillow where it reacts with the pillow cores^37^. These interactions result in different geochemical signatures for the inner pillow basalt and their glass rims^38^,^39^,^40^.

Large Ion Litophile Elements behave similarly during hydrothermal alteration and during melting processes, that is, they partition strongly into the fluid phase. Hence, alkali elements like Rb, Sr, K, Mg, Ca, Na and Ba show variations in content from rims to cores in pillow basalts displaying a mobile behaviour. High-field strength elements such as Ti, Th, Zr, Nb and Ta, and to some extent the middle and heavy rare earth elements are less mobile in fluid phases during hydrothermal alteration, which results in their contents remaining unchanged from rims to cores in the pillow basalts^39^. Consequently, pillow glass rims are usually enriched in elements like Fe, Mg, Mn and Rb while showing depletions in Si, Ca, Na, K, Sr and Pb. The latter elements tend to percolate inside the pillow basalt and enrich the core of the pillow basalts^39^. These patterns in element behaviour motivated us to comparatively analyse the core and the glass rims of the pillow basalts from the Nicoya accreted oceanic terranes (Fig. 2e). Peaks in elements like Rb, Ba, K, Pb and Sr show an enriched signature in the pillow cores when performing analyses in bulk rock pillow core and/or rim. However, when the analyses were made *in situ* in the fresh basaltic glasses of the rims an opposite trace element pattern is evident (Fig. 2e). Hence, by analysing the glass rims instead of the bulk pillow sample we can avoid the effect of seawater interaction and obtain a better signature of the original lava composition. This is of most importance given that the pillow core enriched signature can be interpreted as an arc signature when actually it is an artifact of seawater–lava interaction, which can result in misled interpretations of their origin.

**Figure 4:**
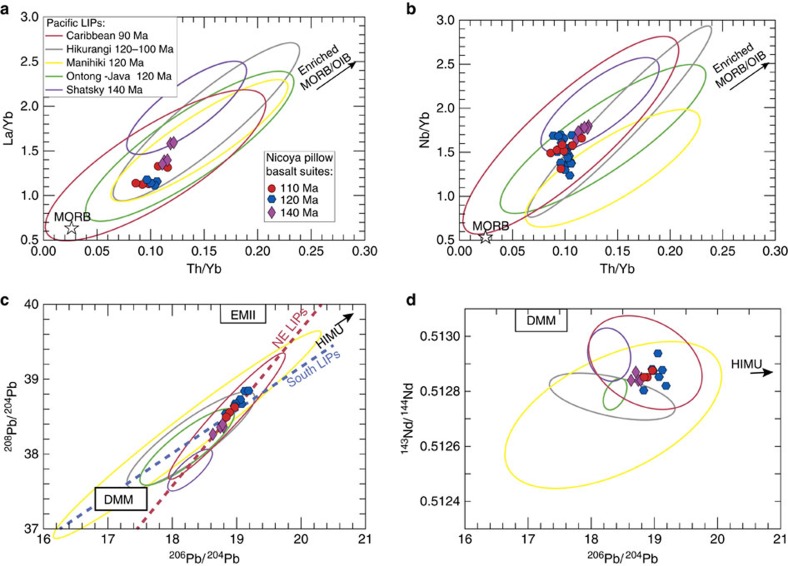
Geochemistry of the Nicoya accreted LIPs compared with contemporaneous Pacific LIPs. Data density ellipses for the Pacific LIPs were calculated at the 95% confidence level. (**a**) La/Yb versus Th/Yb. (**b**) Nb/Yb versus Th/Yb. All the LIP trace element data can be described as the result of different amounts of mixing of an enriched end-member (Enriched MORB/OIB (ref. 59)) and a depleted MORB endmember^60^. (**c**) ^208^Pb/^204^Pb versus ^206^Pb/^204^Pb. (**d**) ^143^Nd/^144^Nd versus ^206^Pb/^204^Pb isotope systematics for the basalts of the different LIPs compared with the accreted terranes of Nicoya I and II showing distinct trends for LIPs originated at the northeastern edges of the LLSVP (Shatsky, Caribbean, Nicoya I and II) and for the southern edge LIPs (Ontong-Java, Manihiki, Hikurangi). All Pacific LIPs show a clear mixing relation between DMM and HIMU end-members, although Manihiki samples also require a third component to explain the low Nd isotopes at a given Pb isotopes.

After a careful assessment of the use of fresh pillow glasses, we applied geochemical constraints to further evaluate the provenance of the Nicoya terranes and their possible relation with other Pacific LIP events. Our samples display flat REE patterns (La/Yb=1.0–1.6) (Fig. 4a), which have been interpreted as the result of the high melt fractions (20–30%) characteristic of the head stage of mantle plumes^7^. Although large melt fractions produced by the upwelling of a hot plume head will result in a homogenization of the melts, the trace element and isotopic compositions of oceanic LIP basalts can also suggest mixing between enriched components ascending within the mantle plume and entrained depleted material. As an example in La/Yb versus Th/Yb space (Fig. 4a), all the data from oceanic LIP basalts (including our new data from the Nicoya terranes) can be explained by mixing between depleted and enriched EMORB/OIB end-members^41^.

**Figure 5:**
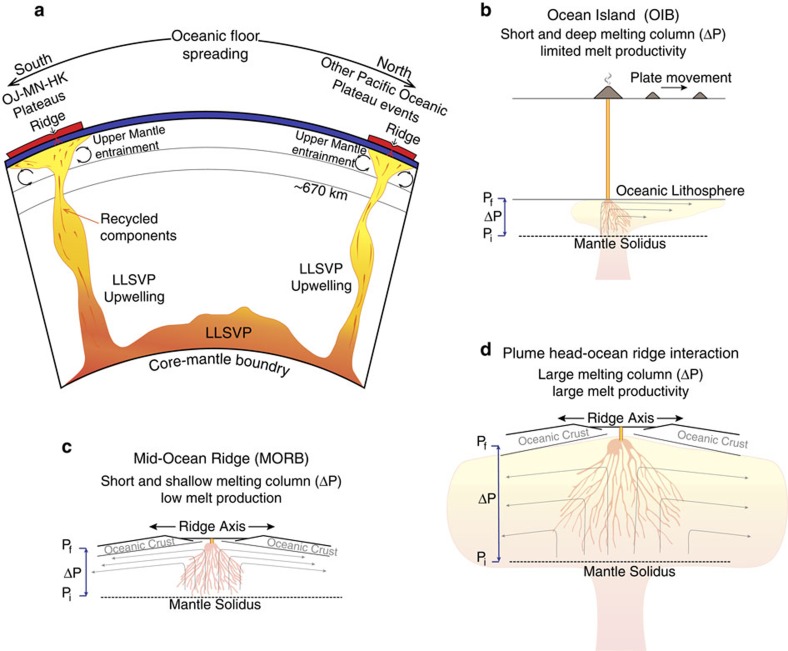
Comparison of melting processes and sources at different melting environments. (**a**) Schematic representation of the Pacific LLSVP. Hot heterogeneous material from the core-mantle boundary (CMB) rises through the mantle as a result of instabilities and positive buoyancy. The ascending material carries the geochemical signature of the stagnant reservoirs at the CMB; ancient subducted oceanic slabs, sub-continental lithosphere or sediments. (**b**) At ocean island chains feed by a mantle plume, initial and final melting pressures (P_i_) will occur at higher depths; however, the melting column (ΔP) will be limited due to lithospheric thickness (P_f_). (**c**) In the case of MORs both P_i_ and P_f_ occur at shallow levels. (**d**) And in the case of a plume head impacting at a MOR a combination of conditions occur where P_i_ will be deep and P_f_ shallow; consequently, the melting column (ΔP) and melt production are large, making the conditions for the emplacement of a LIP ideal.

Along the same line, the radiogenic isotope data from the Pacific LIPs require a mixing relationship between a depleted MORB mantle (DMM) and a HIMU-like mantle reservoir^41^,^42^. Enriched mantle components like HIMU have been attributed to recycling of ancient subducted oceanic lithosphere and carbonated sediments^43^ that persist for billions of years as heterogeneities ponded at the core-mantle boundary^5^ at the location of the LLSVP (Fig. 5a). Once these heterogeneities destabilize and buoyantly ascend they entrain and mix with the surrounding ambient depleted mantle. Lavas from the Pacific LIPs show enrichments towards a HIMU component, when plotted in ^208^Pb/^204^Pb versus ^206^Pb/^204^Pb space (Fig. 4c). The Nicoya I and II terranes seem to share this same isotopic signature. It should also be noted that the samples from the Pacific LIPs erupted at the northeastern edges (that is, Shatsky, Nicoya I and II, and Caribbean) of the LLSVP show a steeper isotopic trend than the values from the southern LIPs (that is, Ontong-Java, Manihiki and Hikurangi) denoting a slight difference in their source compositions (Fig. 4c). This is also evident in the high-field strength elements (for example, Nb/Yb Fig. 4b) systematics as well as in ^143^Nd/^144^Nd versus ^206^Pb/^204^Pb space (Fig. 4d) where Nicoya I and II terranes plot similarly to Shatsky and Caribbean LIPs. Samples from the Manihiki Plateau require another isotopic reservoir (lower in both Nd and Pb isotopes) not evident in the rest of the LIPs. But overall, the trace element and isotopic compositions found in these LIPs are remarkably similar, suggesting that perhaps mantle plumes ascending from the LLSVP are tapping a common deep source^44^ (Fig. 5a).

Our tectonic reconstructions as well as the geochemical signature of these lavas suggest that to produce the Pacific LIPs there was an interaction between upwellings at the edges of the LLSVP and MORs at the surface. Therefore, the major element composition of the primary magmas should be consistent with a deep melt source that also was able to reach relatively shallow levels through a MOR (ref. 7). To test this hypothesis, we updated the work of Herzberg and Gazel^7^ with new modelled primary magmas using PRIMELT3 (ref. 45). Conceptually, the initial melting pressures represent the pressure at which the mantle adiabat crosses the peridotite solidus, and the final melting pressures possibly represent a rheological boundary like the lithosphere–asthenosphere boundary, or in extreme cases the exhaustion of fusible mineral phases^45^ (Fig. 5b–d).

**Figure 6:**
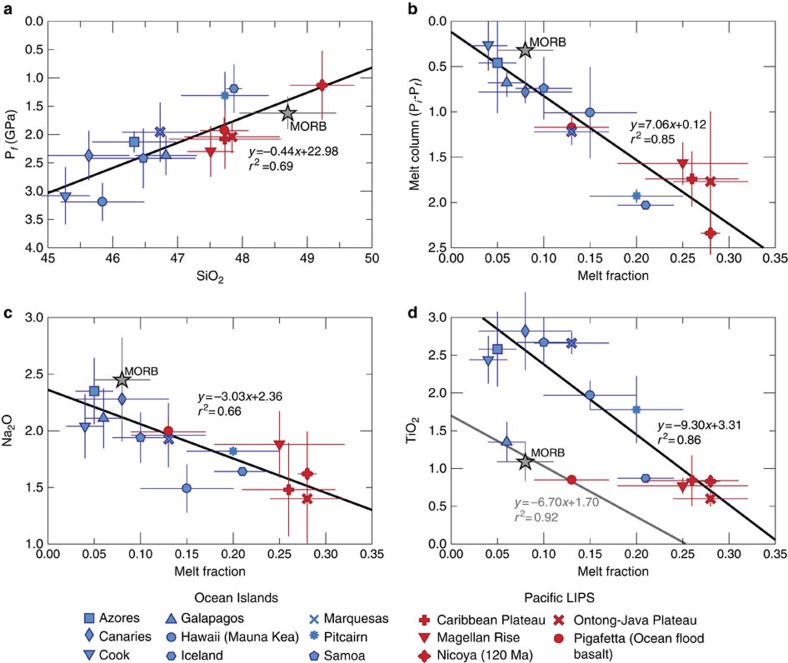
Correlation diagrams showing the different trends for average petrological constraints and primary magma compositions of modern oceanic islands (OIB), LIPs and MORB. (**a**) Final melting pressures (P_f_) plotted against SiO_2_ content. LIP primary magmas are characterized by low pressure values and high SiO_2_ contents, similar to MORB. (**b**) Melt column (P_initial_−P_Final_) plotted against melt fraction; LIPs display more extended melt columns that resulted in higher melt productivity, compared with MORB and OIB. (**c**) Average Na_2_O content versus melt fraction. Note how overall LIPs have the highest melt fractions and lowest Na_2_O contents. (**d**) Average TiO_2_ contents against melt fraction. Two distinct trends can be identified where LIPs show the lowest TiO_2_ contents and the highest melt, but in in the same correlation with most OIB primary magmas. Average MORB, Pigafetta, Galapagos and to some extend Iceland define another trend. The error bars denote one s.d. from the average.

Using the FeO and MgO contents from the calculated primary magmas, we determined the initial and final pressures at which melting occurred^7^,^46^ (Fig. 6). Of particular interest are the average final melting pressure results for LIPs which are between 2.5 and 1.5 GPa, overlapping with modern MORB data; we interpret these results as the interaction between deep mantle upwellings and MORs during the formation of oceanic plateaus. In contrast, in recent ocean island basalt (OIB) locations the average final melting pressures are >2.0 GPa consistent with the upwelling of thermal anomalies beneath a thick lithospheric lid. The two exceptions are Iceland being formed near a ridge^47^ and Pitcairn that formed on top of young oceanic crust^48^. The positive correlation between SiO_2_ (and thus silica activity) of primary magmas and the final melting pressures (Fig. 6a) confirms the fact that LIP melting reached shallow levels^49^. On the other hand, the length of the melting column (as defined by P_initial_−P_final_) is significantly higher for most LIPs compared with modern MORB and OIB data, consistent with the high melt productivity that characterize LIPs (Fig. 6b) due to the excess temperature in their mantle source^7^,^50^. We also found a negative correlation between average Na_2_O contents and melt fraction (Fig. 6c) in agreement with similar correlations previously shown in global MORB systematics^49^,^51^. Finally, we plotted TiO_2_ (a routinely analysed incompatible element) with melt fraction, resulting in two evident trends, one for OIB and LIPs suggesting a more enriched mantle source than for MORB (Fig. 6d), and a second depleted trend that includes MORB along with Galapagos, Pigafetta and partially overlapping with Iceland, which evidence a more depleted mantle component for these primary magmas, consistent with a current interaction with a MOR in these locations^23^,^52^.

### Global impact of upwellings from LLVPs and LIP formation

Our kinematic plate tectonic reconstructions from ∼168 Ma to ∼95 Ma consistently show potential upwellings from the edges of the Pacific LLSVP coincided in the surface with a MOR during the formation of the known Pacific LIPs. These events are separated by time lapses of 10–20 Ma and followed by periods of major oceanic chemistry change and anoxia that cause the disruption of the oceanic biodiversity^53^,^54^. These changes in the ocean are evidenced by major disturbances to the planet's carbon cycle associated with a rise in temperatures and in organic productivity that remain recorded as global oceanic anoxic events (characterized by the widespread formation of black shales). These global oceanic anoxic events are associated with most of the Cretaceous LIPs^55^, and in some cases they can be related with more than one LIP active at the same time (Fig. 1b); this scenario would exacerbate the conditions in the oceans triggering extensive oceanic biota extinctions.

The fact that the bulk emplacement of LIPs (∼120–80 Ma) in the Pacific also coincide with the timing of the Cretaceous Normal Superchron (Fig. 1b), which can be related to fluctuations of mantle-core heat fluxes^56^,^57^, further supports the hypothesis of deep mantle origin for LIPs. Thus, if LIP-producing plumes were rooted in a boundary layer at the base of the mantle (LLSVP) it is possible that instabilities of the buoyant perovskite-bearing peridotite equivalent progressively entrained recycled components^4^,^10^,^58^ making them less buoyant with time and thus explaining the potential cyclicity observed during the Cretaceous. Alternatively, the potential cyclicity of LIP emplacement could also be related to core heat fluctuations interacting with the lower mantle, pulses of material crossing the transition zone (either upwelling hot material or downgoing dense slabs), or a combination of both processes. Even though these hypotheses require further evaluation, recognizing patterns and possible cycles is crucial to the link between deep processes and life. The Pacific Plate preserves the evidence for deep mantle upwellings in the Cretaceous but just as the accreted fragments found in Costa Rica, many other unrecognized LIP events could be preserved accreted along the margins of the Pacific Ocean. Tying these events to upwellings of long-lived anomalies such as the Pacific LLSVP can help us understand the Earth's interior processes that happened in the past and the potential for future catastrophic LIP activity.

## Methods

### Geochronology and geochemistry methods

Fresh basaltic glasses from the hyaloclastite and pillow rims were collected from the Murcielago Islands and the Nicoya Peninsula. GPS locations are reported in the Supplementary Data 1. These samples were carefully selected and cleaned to get the fresher pieces of glass. Glass chips of 425–300 μm in diameter were obtained by dry-sieving.

To acquire the ^40^Ar/^39^Ar data, the groundmass and mineral separates were irradiated for 60 h at the Oregon State University TRIGA-type reactor in the Cadmium-Lined In-Core Irradiation Tube. At the University of Wisconsin–Madison Rare Gas Geochronology Laboratory, incremental heating experiments were conducted using a 25 Watt CO_2_ laser. Each step of the experiment included heating at a given laser power, followed by an additional 10 min for gas cleanup. The gas was cleaned with two SAES C50 getters, one of which was operated at ∼450 °C and the other at room temperature. Blanks were analysed after every second laser heating step, and were <5 × 10^−20^ mol V^−1^ for ^36^Ar and 2 × 10^−17^ mol V^−1^ for ^40^Ar, respectively. Argon isotope analyses were performed using a MAP 215–50, and the isotope data were reduced using ArArCalc software version 2.5 (http://earthref.org/ArArCALC/). Ages were calculated from the blank-discrimination and decay-corrected Ar isotope data after correction for interfering isotopes produced from potassium and calcium in the nuclear reactor (Supplementary Data 2–6). Ages are reported with 2σ uncertainties (includes the J uncertainty) and are calculated relative to a Fish Canyon standard age of 28.201±0.046 Ma (ref. 61) and a value for λ^40^K of 5.463±0.107 × 10^−10^ per year^62^.

Basaltic glass samples collected from the Murcielago Islands and Nicoya Peninsula pillow basalts rims were selected under a stereoscope microscope, and arranged in a 1-inch round epoxy mount which was later polished for electron microprobe analyses. Major element data were collected at the Electron Beam Laboratory at Virginia Tech with a Cameca SX50 Electron Microprobe using a 60 μm diameter electron beam at a 10 nA current a 15 kV acceleration voltage. Trace elements were obtained at Virginia Tech LA-ICPMS lab facilities using an Agilent 7500ce ICPMS coupled with a Geolas laser ablation system. Three analyses were performed in each glass using a 90 μm diameter spot and at 10 Hz repetition rate. Standards were run at the start and end of the run to correct for drift. The data were reduced using the USGS standards BCR-2G, BHVO-2G and BIR-1G. Replicates of these standards indicate a precision of <5% (relative standard deviation (RSD)) and accuracy better than 10% for most of the elements analysed (Supplementary Data 1). Analysis of Sr, Nd and Pb radiogenic isotope ratios were carried out at the Center for Elemental Mass Spectrometry, University of South Carolina following established techniques for this lab^63^. Major and trace element geochemistry along with isotopic data are reported in Supplementary Data 1. To guarantee an acceptable quality in the data, we chose only the samples that yielded an ^40^Ar/^39^Ar plateau age (with ^39^Ar% higher than 50%) or samples that generated inverse isochrones ages for our study. Also, we used only data obtained by step-heating techniques to avoid anomalous sub-systems within the measurements due to Ar loss or inherited Ar. Previous studies have published K-Ar ages for the Nicoya Complex oceanic rocks; however, these ages are not included in our compilation because the low potassium contents of the basalt suites and the varying degrees of secondary seafloor alteration introduce uncertainty to the measurements. Altered samples can be subject of partial loss of radiogenic ^40^Ar derived from *in situ* decay of ^40^K which can lead to an under/over estimation of the crystallization ages. Supplementary Table 1 includes the ^40^Ar/^39^Ar geochronological analyses from the Nicoya Complex reported in previous works by Sinton *et al*.^64^, Hauff *et al*.^14^ and Hoernle *et al*.^15^. In this work we present five new ages from El Coco (137.09±2.48 Ma), Murcielago Islands (113.43±3.48 Ma), inner Nicoya (89.3±4.3 Ma), Junquillal (79.9±0.7 Ma) and Marbella (77.2±2.7 Ma); the detailed step-heating experiments and ^40^Ar/^39^Ar spectra are available in the Supplementary Data 2–6. The ages from El Coco and Murcielago Islands were measured in fresh pillow basalt glasses and they belong to the Nicoya I and II LIP pulses. Samples from Junquillal, Marbella and inner Nicoya are from diabases and belong to intrusive events related to the main and subsequent pulses of the Caribbean LIP. Additional ^40^Ar/^39^Ar ages for other LIP events compiled from the literature are in the Supplementary Table 2 and plotted in Fig. 1a.

### Kinematic plate tectonic reconstruction parameters

Our new kinematic plate tectonic model is based on data derived from different geological, geodynamic and kinematic constraints. Besides our own geological investigations in Central America, the distribution, composition, age and evolution of the continental blocks, magmatic provinces, suture zones, sedimentary basin and accreted complexes in Middle America region have been systematically compiled in a geo-referenced database^65^. Our model differs from earlier models because we integrated dynamic plate boundaries, plate buoyancy factors, oceanic spreading rates, subsidence patterns, stratigraphy and paleobiogeographic data, as well as the major tectonic and magmatic events. Our new plate tectonic models also combine the classically used Atlantic Ocean constraints (Pangea breakup) with the geodynamic history of the Americas active margins to place and reconstruct the Pacific Ocean plates. This new approach represents a distinct departure from classical continental drift models, which only consider displacement of continents, terranes and blocks on a sphere and do not take into account plate boundaries and oceanic crust geometry. The kinematic models presented here match isochrones 5o and 6o on the Pacific, Antarctica, Nazca and Cocos plates (Supplementary Fig. 2). Asymmetric counterpart isochrones for 13y, 18o, 21o, 25y, 31y and 34y were constructed on the Farallon Plate seafloor from the Oligocene to the Campanian (for example, Fig. 3i–k). A combination of using fixed Atlantic and Pacific hotspots as a framework to calculate relative plate motions, and interpreting the onshore geology such as subduction complexes, accretionary terranes, ophiolites and larger-scale crustal deformation approaches were used to reconstruct isochrones during the Cretaceous Normal Superchron (Fig. 3f–h), as well as to reconstruct counterpart isochrones for M0, M10, M16, M25 and M42 (Fig. 3a–e). Finally, isochrones M21 and M4 were used as kinematic constraints for the geometric evolution of the Pacific Plate during the Late Jurassic to Early Cretaceous.

The new plate tectonic results presented here (Fig. 2 and Supplementary Figs 3–5) were created using a geological-geodynamic approach first explained and applied by Stampfli and Borel^66^. The reconstructions were performed using an ArcGis base, which enabled us to apply and quantify rotational motions and spreading/subduction rates to the numerous plates and tectonic blocks involved in the evolution of the study area. Boundary conditions are provided by the relative motions of the different plates with respect to a fixed Europe (Baltica). Plate tectonic concepts are applied all along the process and plate boundaries are built and transformed in space and time. Plate velocities can be calculated at any time and are never in excess of 20 cm per year. The reconstructions were created from the past to the present, although an iterative approach is always necessary. The size of the ancient oceanic domains was created using geometric constraints done by geometry of Pacific and Atlantic oceans magnetic isochrones see (Supplementary Fig. 4; ref. 67) and by the ages of collision events recorded in the Middle American region^68^,^69^. The lithospheric plates were constructed through time by adding/removing oceanic crust to the major continents and terranes. Plates were created systematically using tight fits in order not to underestimate crustal extension. Each plate was moved step by step, as single rigid entities. The only evolving elements are the plate boundaries, which are preserved and follow a consistent geodynamic evolution through time. This methodology offers us a good control on plate kinematics and geometries, which provide new constraints for plate tectonic scenarios and their relationship with the geological record.

Due to the lack of magnetic lineations during the Cretaceous Normal Superchron, we created three kinematic plate tectonic reconstructions at 112, 103 and 95 Ma (Supplementary Fig. 3f–h) using reconstructed isochrones at symmetries and spreading rates that would provide a seafloor geometry to satisfactory explain collisions and other major geological and tectonic events recorded on the Middle America active margin. At the 112 Ma (Supplementary Fig. 3f), we propose a new ridge triple point formation in the southern Pacific Plate MOR, which connected the Pacific Plate with a new southeastern oriented ridge that opened perpendicular to Phoenix Plate seafloor. This triple point allowed the separation of the Manihiki and Hikurangi from the Ontong-Java Plateau.

Data and methodology applied here are part of a larger global geodynamic database created to support plate tectonic reconstruction extending from the Late Neoproterozoic to the Cenozoic. Examples of this new approach can be found in Hochard^70^, whole globe; Bagheri and Stampfli^71^, Iran; Moix, *et al*.^72^, Turkey; Ferrari, *et al*.^73^, Southeast Asia; von Raumer and Stampfli^74^, Rheic Ocean; Flores^65^, Central America; Stampfli and Hochard^75^, Alpine realm; Vérard, *et al*.^76^, South America-Antarctica; Wilhem, *et al*.^77^, Altaids; Vérard, *et al*.^78^, Global Euler poles distribution; Vérard and Stampfli^79^, Australides; and Stampfli, *et al*.^80^, Pangea. Our kinematic reconstructions do not significantly differ from those on Müller, *et al*.^25^, (Supplementary Fig. 4) which integrated the absolute plate motion models. In our reconstructions, the Triangle of Pacific plate display four different rotational paths inside the LLSVP edges (Supplementary Fig. 5a), initially a clear south migration is observed from ∼168 Ma to ∼132 Ma (Supplementary Fig. 5b). A small step backwards is followed by a steady SW rotation occur from ∼120 Ma to ∼103 Ma (Supplementary Fig. 5c). A ∼10 Ma reversal period of rotation towards the NE is observed (Supplementary Fig. 5d) before its final NW migration recorded from ∼68 Ma to the present (Supplementary Fig. 5e). These rotational paths can be correlated to the growth, migration and collision of the various island arcs that surrounded the Pacific plate since ∼168 Ma to ∼84 as well as the establishment of a continued eastward subduction zone along the Americas after the formation of the modern Caribbean plate around 68 Ma.

### Data availability

All data pertinent to this manuscript can be found in the manuscript itself or the associated Supplementary Information file.

## Additional information

**How to cite this article**: Madrigal, P. *et al*. Record of massive upwellings from the Pacific large low shear velocity province. *Nat. Commun.* **7**, 13309 doi: 10.1038/ncomms13309 (2016).

**Publisher's note:** Springer Nature remains neutral with regard to jurisdictional claims in published maps and institutional affiliations.

## Supplementary Material

Supplementary Information Supplementary Figures 1-5, Supplementary Tables 1-2, Supplementary Note 1 and Supplementary References

Supplementary Data 1 New major elements, trace elements, and isotope geochemistry for the Nicoya accreted terranes, standard replicates, primary magma calculations for Pacific LIPs, and LIPs age compilation.

Supplementary Data 2 Detailed step-heating experiments and 40Ar/39Ar spectra 1

Supplementary Data 3 Detailed step-heating experiments and 40Ar/39Ar spectra 2

Supplementary Data 4 Detailed step-heating experiments and 40Ar/39Ar spectra 3

Supplementary Data 5 Detailed step-heating experiments and 40Ar/39Ar spectra 4

Supplementary Data 6 Detailed step-heating experiments and 40Ar/39Ar spectra 5
