# Supplementary material for: Record of massive upwellings from the Pacific large low shear velocity province
Source: Nat Commun. 2016 Nov 8;7:13309. doi: 10.1038/ncomms13309 (PMC5105175; doi:10.1038/ncomms13309)
Supplement: Supplementary Information — Supplementary Figures 1-5, Supplementary Tables 1-2, Supplementary Note 1 and Supplementary References [file ncomms13309-s1.pdf]

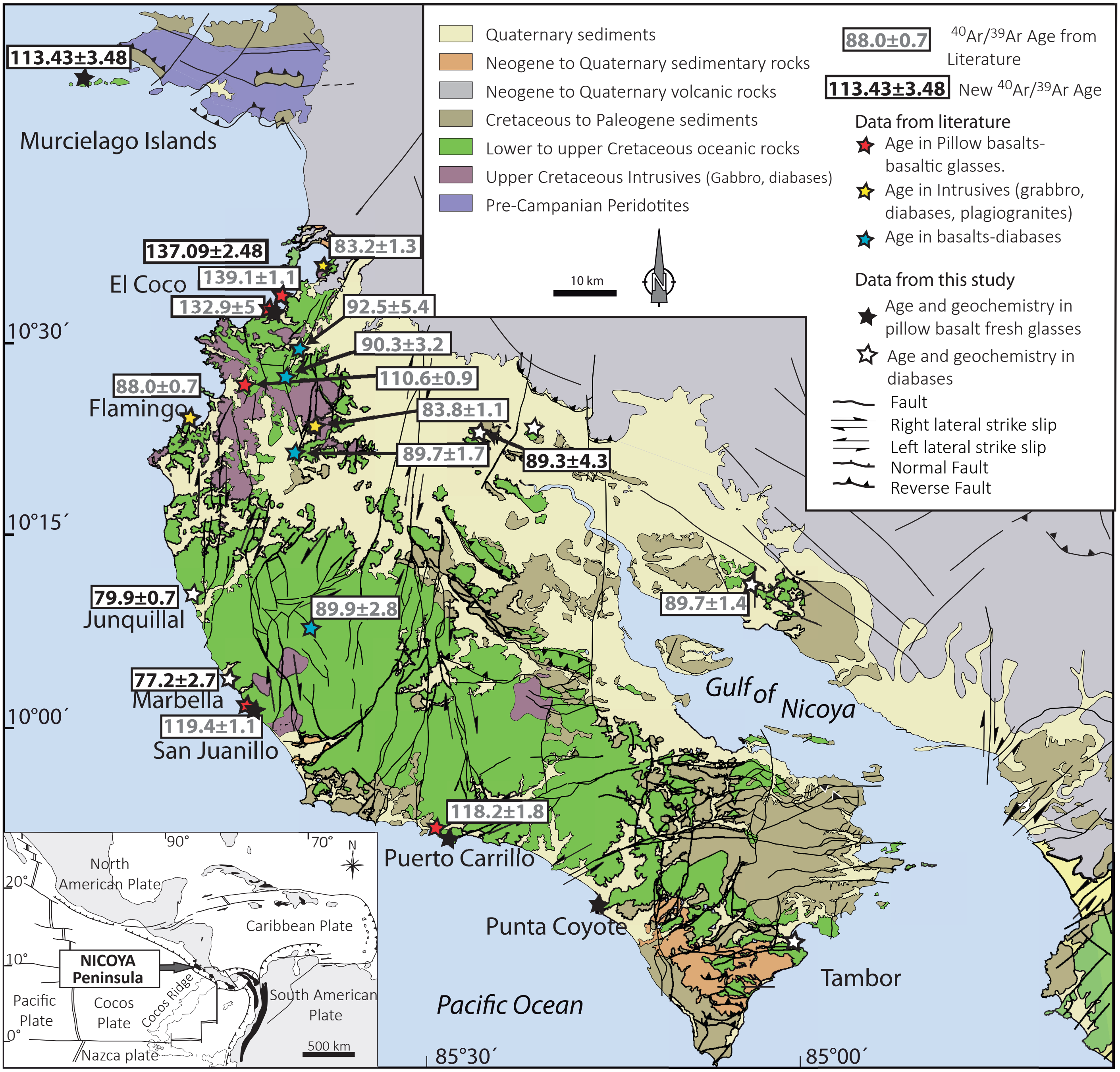

Supplementary Figure 1: Geologic map of the Nicoya Peninsula Costa Rica, modified after Denyer, et al[1]. Samples collected and analyzed for this study are shown in black star symbols for pillow basalt fresh glasses and in whitestar symbols corresponding to diabases. Samples from the literature use the following symbols: red stars for pillow basalt and basaltic glass samples, yellow stars for ages in intrusive samples (gabbro, diabase or plagiogranites) and blue for samples described in the literature as coarse-to-fine grain basalt and that can correspond to massive flows or diabases. $^{40}Ar/^{39}Ar$ ages from the literature are shown in white boxes with gray font and our new $^{40}Ar/^{39}Ar$ analyses are in black fonts. Inset on the left shows the location of the Nicoya Peninsula within the geotectonic frame of the region. All ages are in millions of years (Ma).

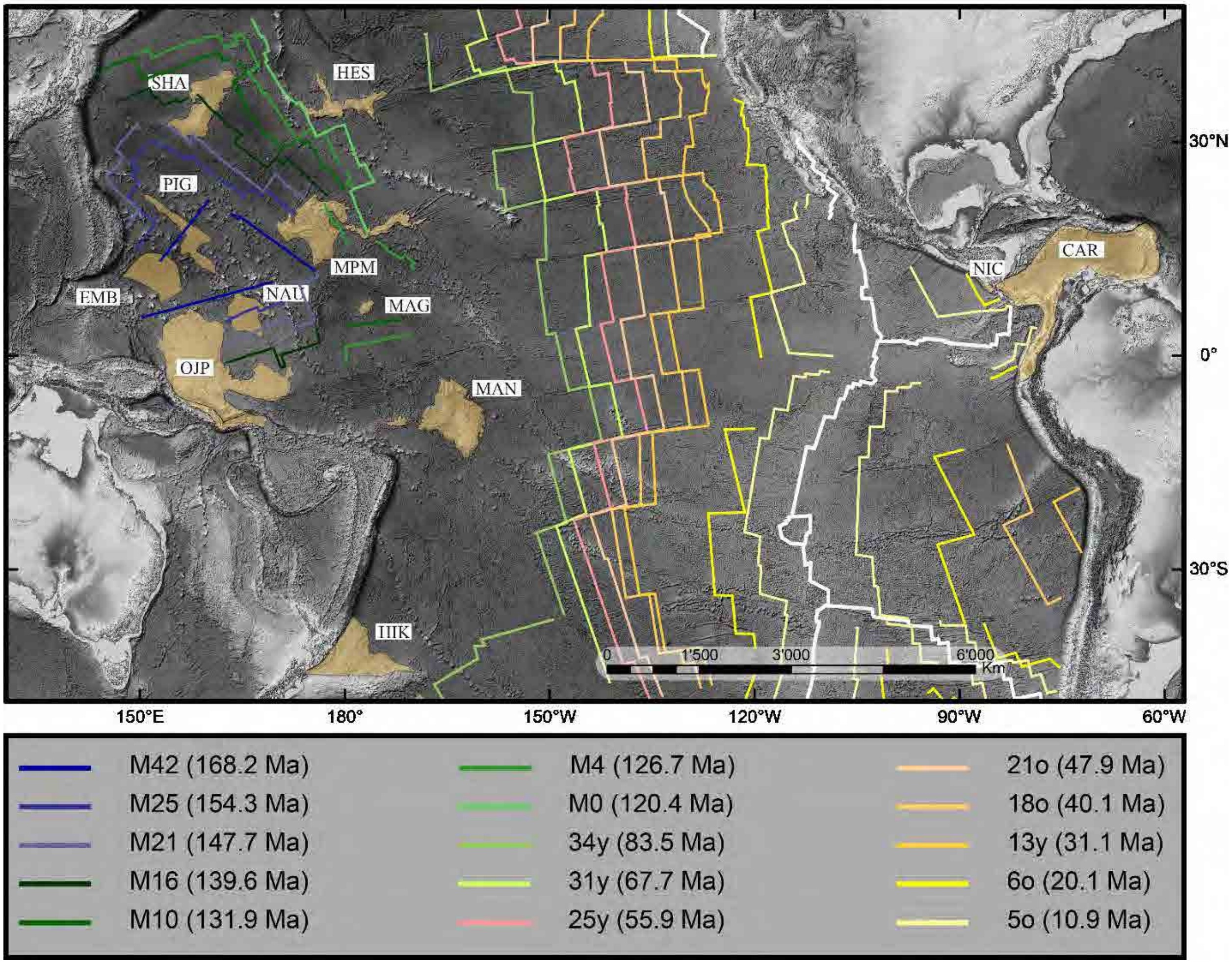

Supplementary Figure 2. Central Pacific Ocean seafloor spreading map, the color lines represent the isochrones used for our kinematic model. Letters, numbers and ages correspond to magnetic isochrons after Müller, et al.[17] and Müller, et al.[18].

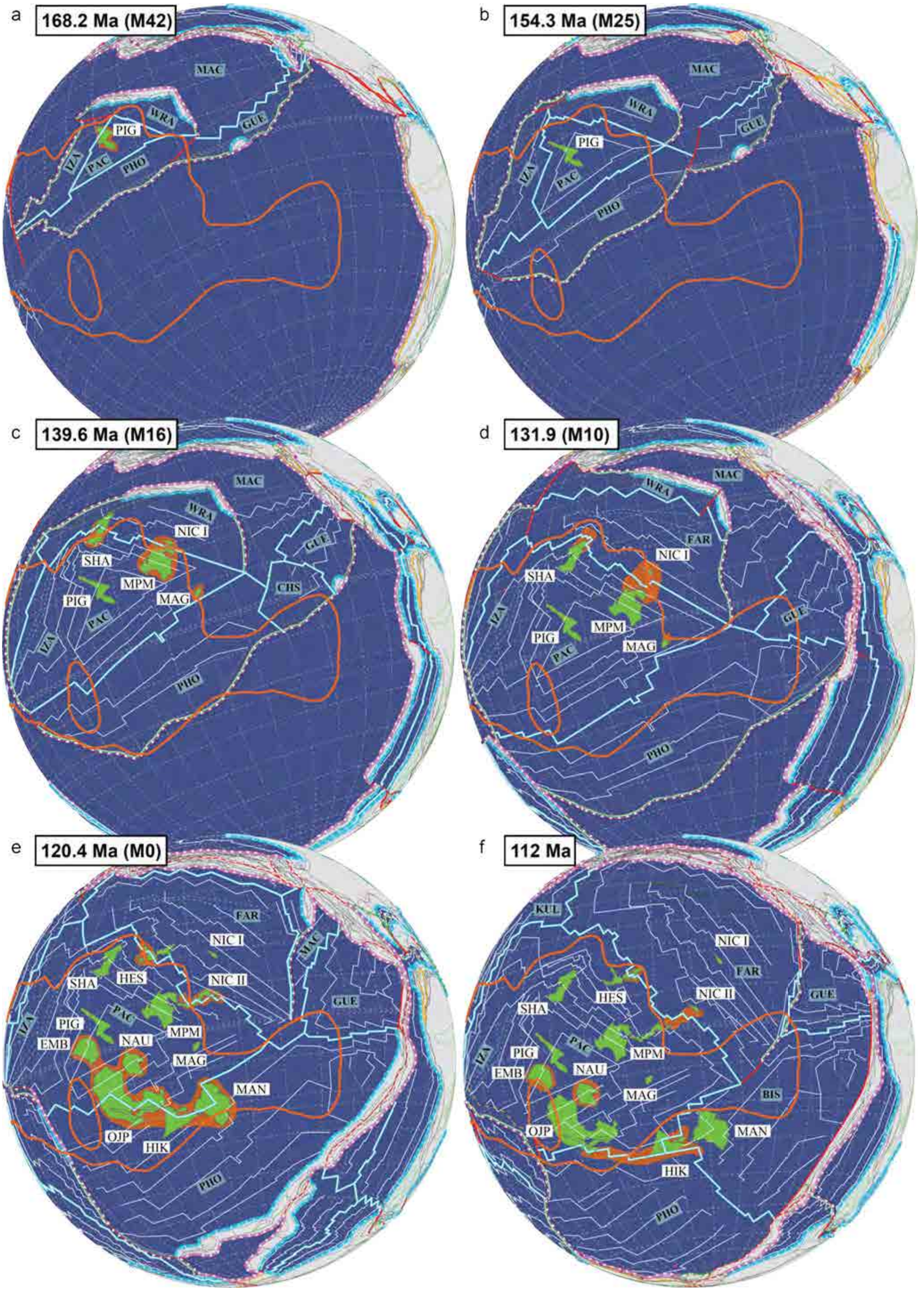
a
168.2 Ma (M42)
MAC
WRA
GUE
PIG
IZA
PAC
PHO
b
154.3 Ma (M25)
MAC
WRA
GUE
PIG
IZA
PAC
PHO
c
139.6 Ma (M16)
MAC
WRA
NIC I
GUE
SHA
MPM
CHS
PIG
MAG
PAC
IZA
PHO
d
131.9 (M10)
MAC
WRA
FAR
NIC I
SHA
IZA
MPM
PIG
GUE
PAC
MAG
PHO
e
120.4 Ma (M0)
FAR
NIC I
MAC
SHA
HES
NIC II
GUE
PAC
PIG
IZA
MPM
EMB
NAU
MAG
MAN
OJP
HIK
PHO
f
112 Ma
KUL
NIC I
FAR
HES
NIC II
GUE
SHA
IZA
PAC
MPM
PIG
EMB
NAU
MAG
BIS
OJP
MAN
HIK
PHO

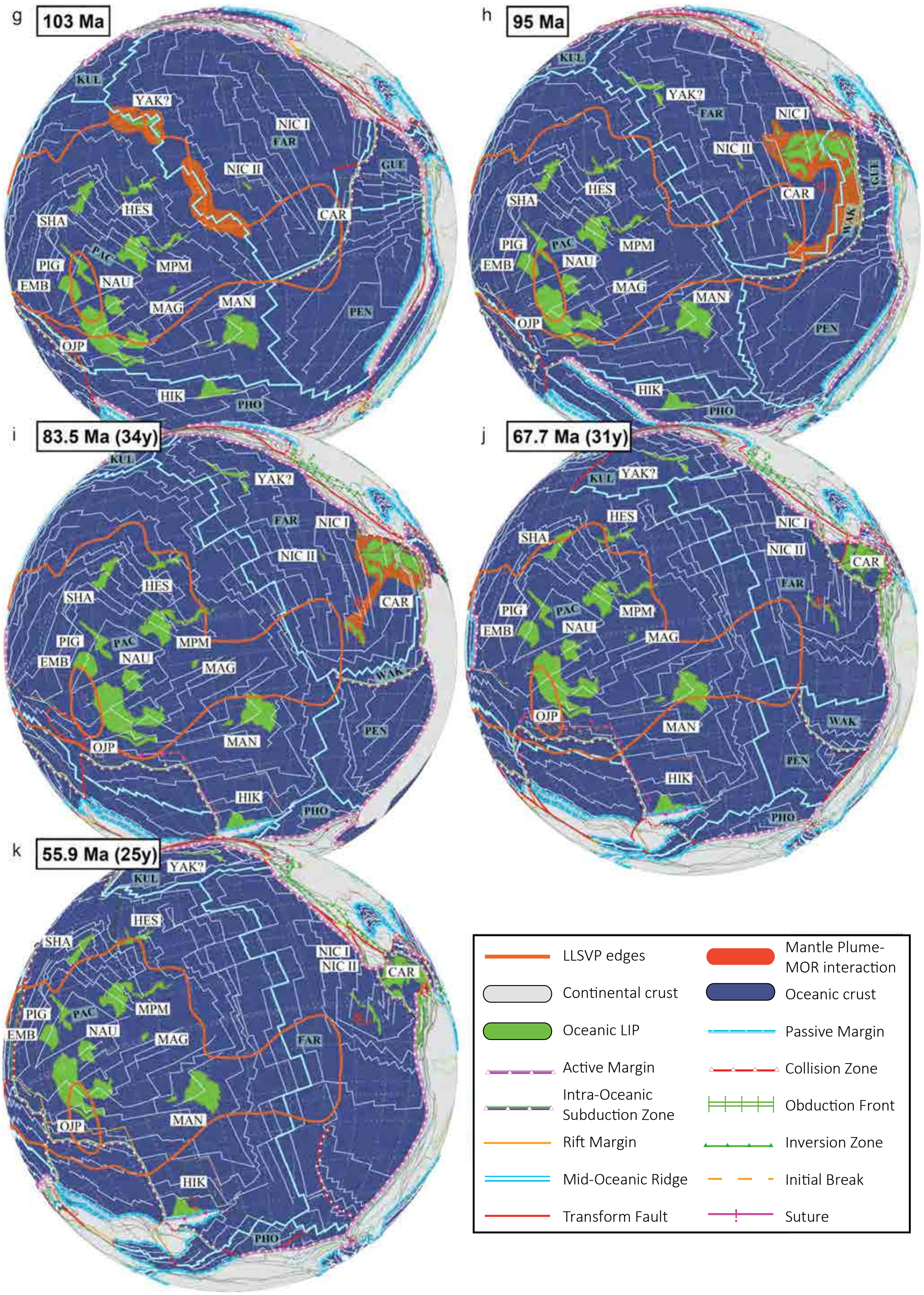

Supplementary Figure 3. Reconstructions at a-168.2 Ma (M42), b- 154.3 Ma (M25), c- 139.6 Ma (M16), d-131.9 Ma (M10), e- 120.4 Ma (M0), f- 112 Ma, g- 103 Ma, h- 95 Ma, i- 83.5 Ma (34y), j-67.7 Ma (31y) and k-55.9 Ma (25y). Notations in parenthesis refer to established magnetic anomalies. Tectonic plate abbreviations BIS (Biscoe), CHS (Chonos), FAR (Farallon), GUE (Guerrero), IZA (Izanagi), KUL (Kula), MAC (Mackinley), PAC (Pacific), PEN (Penas), PHO (Phoenix), WAK (Washikemba), WRA (Wrangellia). See text for further descriptions. LIP abbreviations same as in the main text.

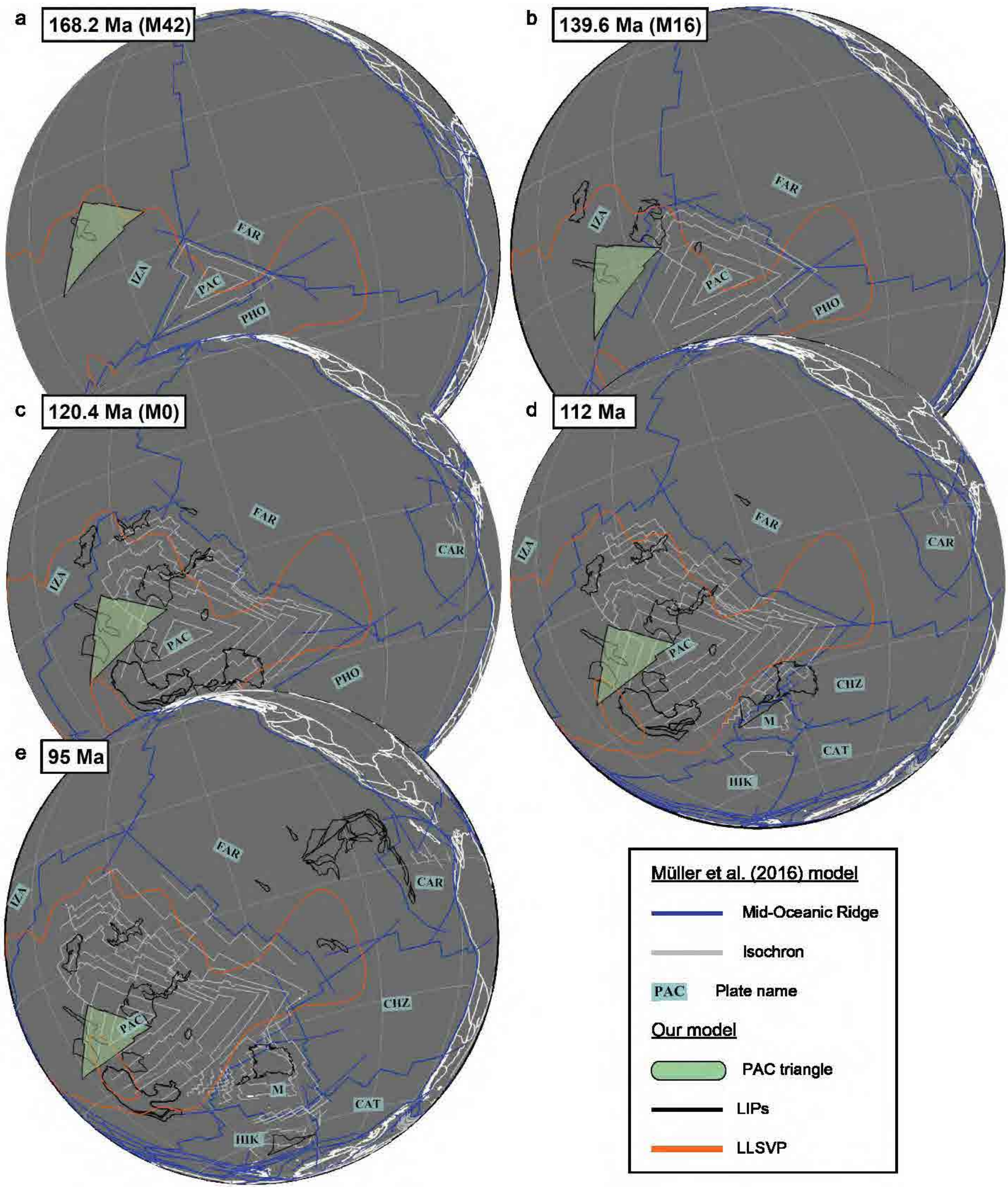

Supplementary Figure 4. GPlates-based plate tectonic reconstructions after Müller, et al.[17] compared to the positions of the LLSVP edge, Large Igneous provinces (LIPs), and the Pacific plate triangle given by our kinematic reconstructions. Note the both models do not dramatically differ on the position of the Pacific plate for the given times. Tectonic plate abbreviations from Müller, et al.25 CAR (Caribbean), CAT (Catequil), CHZ (Chazca), FAR (Farallon), HIK (Hikurangi), IZA (Izanagi), M (Manihiki), PAC (Pacific), and PHO (Phoenix).

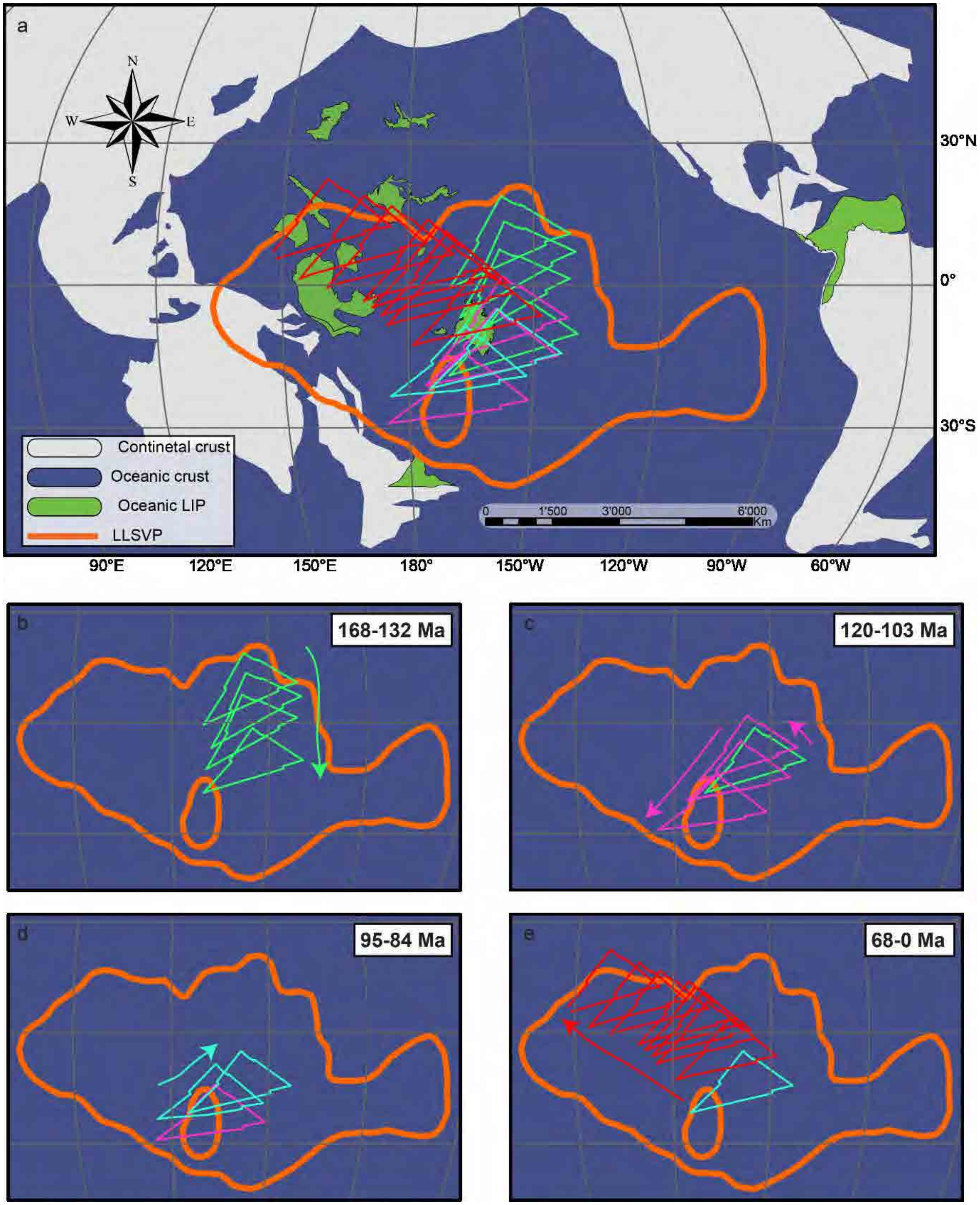

Supplementary Figure 5. Rotational evolution of the Pacific plate triangle based on our kinematic plate tectonic model. Note how the migration paths describe a relative motion towards the south at ~168-132 Ma, changing towards southwest at ~120-103 Ma, then towards northwest at ~95-84 Ma and finally to the current northwest rotational path at ~68-0 Ma.

Supplementary Table 1: Geochronological compilation of Nicoya Complex

Sample	Lat/Long	Material	$^{40}Ar/^{39}Ar$ Age Ma	Error	$\%^{39}Ar$ used	Author
**NI-7**	10.5687/ -85.6947	Glass	137.09	2.48	94	This study
**SE-050611-11**	10.8575/ -85.9462	Glass	113.43	3.48	84	This study
**NI-4**	10.3981/- 85.4596	Groundmass	89.25	4.28	88	This study
**NI-12**	10.1768/ -85.8195	Groundmass	79.9	0.77	84	This study
**NI-14**	10.0685/- 85.7717	Groundmass	77.15	2.65	85	This study
**AN34**	10.03161/ - 85.74218	Glass	118	5	64	Hoernle, et al. [7]
**AN10**	10.5693/ -85.6939	Glass	137	2	79	Hoernle, et al. [7]
**AN8**	10.5684/ - 85.69572	Glass	139	1		Hoernle, et al. [7]
**AN3**	10.5570/ -85.71303	Glass	133	1.5		Hoernle, et al. [7]
**AN40**	9.863107/ - 85.487	Glass	118.2	1.8		Hoernle, et al. [7]
**BN22**	10.46514/ - 85.74825	Matrix	110.6	0.9		Hoernle, et al. [7]
**NC93-2**	10.47443/ - 85.69074	Whole rock	90.3	3.2	100	Sinton, et al. [3]
**NC93-3**	10.51340/ - 85.67081	Whole rock	92.5	5.4	100	Sinton, et al. [3]
**NC93-18**	10.36422/ - 85.6692	Whole rock	89.7	1.7	100	Sinton, et al. [3]
**NC93-20**	10.12206/ - 85.62990	Whole rock	89.9	2.8		Sinton, et al. [3]
**NC93-34**	10.41056/ - 85.82198	Whole rock	88	0.7		Sinton, et al. [3]
**NC93-14B**	10.39849/ -85.66206	Plagioclase	83.8	1.1		Sinton, et al. [3]
**NC93-26**	10.60185/ - 85.6494	Plagioclase	83.2	1.3		Sinton, et al. [3]

Supplementary Table 2: Geochronological summary of the Pacific LLSVP upwellings, LIP occurrence and correlation with the Nicoya Accreted terranes

Nicoya terranes	LIP stage	Age range (Ma)	Volume [26]	Age of OIB-rejuvenated stage (Ma)		Proposed LLSVP upwelling
None	Pigafetta Basin	171.5±1.1<br>158.6±2.7	2.0 x 10^5 km^3	127±1.7<br>124.8±0.7		~170-160 Ma
Nicoya I	Shatsky Rise	144.8±1.2	4.3 x 10^6 km^3	?	120-110 Ma	~140 Ma
	MPM basement	140 Ma	42.94 x 10^6 km^3	Mid-Pacific M.		
	Magellan Rise	135 Ma	3.64 x 10^6 km^3	---	---	
Nicoya II	Ontong Java	122.9±1.5	1.01 x 10^8 km^3	OJP Seamounts	90 Ma	~120-110 Ma
	Manihiki	123.8±0.8	1.04 x 10^7 km^3	Manihiki Seamounts	81.6 – 75.1 Ma	
	Hikurangi	118.4±4	2.8 x 10^6 km^3	Hikurangi Seamounts and Guyots	98.7 ± 0.7 Ma<br>87.5 ± 0.4 Ma	
	Nauru Basin	120 Ma	2.0 x 10^5 km^3	Seamounts	?	
	East Mariana Basin	118.3±1.6	0.25 x 10^6 km^3	?	?	
Nicoya III (Caribbean)	Caribbean	90 Ma	20.41 x 10^6 km^3	Cocos Seamounts	~70 Ma	~90 Ma
	Hess Rise	87 Ma	7.78 x 10^6 km^3	?	?	

Supplementary Note 1

The Nicoya Complex geotectonic background

The Nicoya Complex in northwestern Pacific coast of Costa Rica (Fig. 1) includes a series of oceanic terranes of Pacific origin that range ~170 Ma to ~80 Ma[1] (see summary in Fig. 1). Geological studies of the Nicoya Complex exist from the early 1900's; however, its formal definition was made in the 1960s by Dengo [2]. It groups a series of terranes of oceanic origin that comprise of basaltic rocks, gabbros and plagiogranites interlayered with deep sea sediments (radiolarian cherts) of Pacific origin [3-6]. For this study we focused on the suites of pillow basalts that are exposed along the coasts of the Nicoya Peninsula and in the Murcielago Islands (Fig. 1). At these outcrops, pillow basalt flows can be found in excellent state of preservation, including fresh glass rims and inter-pillow hyaloclastite (see main text).

The origin of these oceanic accreted terranes has been related to an early Galapagos mantle plume initiation [3,7,8], accretion of Pacific MORB crust [9,10], accretion of Galapagos hotspot tracks [11,12], uplift of Caribbean oceanic plateau crust [13]. However, the most accepted models for the Nicoya are the ones that involve a pacific origin as part of the Caribbean Large Igneous Province (CLIP) [Denyer and Baumgartner [6]. In their model different magmatic pulses disrupted and detached from the Jurassic-Cretaceous oceanic basement along with the overlying sedimentary sequences as they erupted, resulting in the older oceanic crust and radiolarian cherts embedded within the Caribbean LIP as "xenoliths".

Geochemical data from the Nicoya igneous complex clearly indicates intraplate origin; however, many authors proposed an oceanic island arc affinity [14-16] but those interpretations were based on whole rock data that was influenced by ocean floor alteration (see main text). More recent geochemical analyses linked these mafic igneous suites to the Caribbean LIP event as the samples are characterized by flat rare earth element (REE) patterns, moderate high field

strength element (HFSE) enrichments and common radiogenic isotopic signatures [3,7,11,12]. In this study we present five new $^{40}Ar/^{39}Ar$ ages, 35 new geochemical analyses including major and trace element data and 12 new radiogenic isotope measurements from these Nicoya accreted terranes (Supplementary Dataset 1-6).

References

1 Denyer, P., Aguilar, T. & Montero, W. Cartografia geologica de la peninsula de Nicoya, Costa Rica: estratigrafia y tectonica. 1. edn, (Editorial UCR, 2014).

2 Dengo, G. Tectonic igneous sequence in Costa Rica. In: Engel, A. E. J., James, H. J., Leonard, B. F. (Eds.), A volume to honor A. F. Budington. Geological Society of America Special Volume, pp. 133-161, (1962).

3 Sinton, C. W., Duncan, R. A. & Denyer, P. Nicoya Peninsula, Costa Rica: A single suite of Caribbean oceanic plateau magmas. Journal of Geophysical Research: Solid Earth 102, 15507-15520, (1997).

4 Baumgartner, P., Flores, K., Bandini, A., Girault, F. & Cruz, D. Upper Triassic to Cretaceous radiolaria from Nicaragua and northern Costa Rica: The Mesquito composite oceanic terrane. Ofioliti 33, 1-19, (2008).

5 Baumgartner, P. & Denyer, P. Evidende for middle Cretaceous accretion at Santa Elena Peninsula (Santa Rosa Accretionary Complex), Costa Rica. Geologica Acta 4, 179-191, (2006).

6 Denyer, P. & Baumgartner, P. O. Emplacement of Jurassic-Lower Cretaceous radiolarites of the Nicoya Complex (Costa Rica). Geologica acta 4, 203, (2006).

7 Hoernle, K., Hauff, F. & van den Bogaard, P. 70 m.y. history (136-69 Ma) for the Caribbean Large Igneous Province. Geological Society of America 32, 697-700, (2004).

8 Denyer & Gazel, E. The Costa Rican Jurassic to Miocene oceanic complexes: Origin, tectonics and relations. Journal of South American Earth Sciences 28, 429-442, (2009).

9 Galli-Olivier, C. Ophiolite and island-arc volcanism in Costa Rica. Geological Society of America Bulletin 90, 444-452, (1979).

10 Kuijpers, E. P. The geologic history of the Nicoya Ophiolite Complex, Costa Rica, and its geotectonic significance. Tectonophysics 68, 233-255, (1980).

11 Hauff, F., Hoernle, K., Schmincke, H.-U. & Werner, R. A Mid Cretaceous origin for the Galápagos hotspot: volcanological, petrologica and geochemical evidence from Costa Rican oceanic crustal segments. Geologische Rundschau 86, 141-155, (1997).

12 Hauff, F., Hoernle, K., van den Bogaard, P., Alvarado, G. & Garbe-Schönberg, D. Age and geochemistry of basaltic complexes in western Costa Rica: Contributions to the geotectonic evolution of Central America. Geochemistry Geophysics Geosystems 1, (2000).

13 Duncan, R. A. & Hargraves, R. B. Plate tectonic evolution of the Caribbean region in the mantle reference frame. Geological Society of America Memoirs 162, 81-94, (1984).

14 Wildberg, H. G. H. Der Nicoya Komplex, Costa Rica, Zentralamerika: Magmatismus und genese eines polymagmatischen Ophiolith-Komplexes. Münster Forschngsschwerpunkte Geologisch Paläontologisches 62, 1-123, (1984).

15 Frisch, W., Meschede, M. & Sick, M. Origin of the Central American ophiolites: Evidence from paleomagnetic results. Geological Society of America Bulletin 104, 1301-1314, (1992).

16 Meschede, M. & Frisch, W. Geochemical characteristics of basaltic rocks from the Central American ophiolites. Profil 7, 71-85, (1994).

17 Müller, R. D. *et al.* Ocean Basin Evolution and Global-Scale Plate Reorganization Events Since Pangea Breakup. *Annual Review of Earth and Planetary Sciences* **44**, 107-138, (2016.)

18 Müller, R. D., Sdrolias, M., Gaina, C. & Roest, W. R. Age, spreading rates, and spreading asymmetry of the world's ocean crust. *Geochemistry, Geophysics, Geosystems* **9**, Q04006, (2008).
